# Comparative Analysis of Two *Candida parapsilosis* Isolates Originating from the Same Patient Harbouring the Y132F and R398I Mutations in the *ERG11* Gene

**DOI:** 10.3390/cells12121579

**Published:** 2023-06-07

**Authors:** Matúš Štefánek, Martina Garaiová, Adam Valček, Luisa Jordao, Helena Bujdáková

**Affiliations:** 1 Department of Microbiology and Virology, Faculty of Natural Sciences, Comenius University in Bratislava, 842 15 Bratislava, Slovakia; stefanek8@uniba.sk (M.Š.); valcekam@gmail.com (A.V.); 2 Institute of Animal Biochemistry and Genetics, Centre of Biosciences, Slovak Academy of Sciences, Dúbravska Cesta 9, 840 05 Bratislava, Slovakia; martina.garaiova@savba.sk; 3 Research and Development Unit, Department of Environmental Health, National Institute of Health Dr. Ricardo Jorge, 1649-016 Lisboa, Portugal; maria.jordao@insa.min-saude.pt

**Keywords:** *Candida parapsilosis*, resistance, lipid analysis, fatty acids, *ERG* genes, lipase activity

## Abstract

This work presents a comparative analysis of two clinical isolates of *C. parapsilosis*, isolated from haemoculture (HC) and central venous catheter (CVC). Both strains harboured Y132F and R398I mutations in the gene *ERG11* associated with resistance to fluconazole (FLC). Differences between the HC and CVC isolates were addressed in terms of virulence, resistance to FLC, and lipid distribution. Expression of the *ERG6* and *ERG9* genes, lipid analysis, fatty acid composition, and lipase activity were assessed via qPCR, thin-layer chromatography/high-performance liquid chromatography, gas chromatography, and spectrophotometry, respectively. Regulation of the *ERG6* and *ERG9* genes did not prove any impact on FLC resistance. Analysis of lipid metabolism showed a higher accumulation of lanosterol in both the isolates regardless of FLC presence. Additionally, a decreased level of triacylglycerols (TAG) with an impact on the composition of total fatty acids (FA) was observed for both isolates. The direct impact of the *ERG11* mutations on lipid/FA analysis has not been confirmed. The higher lipase activity observed for *C. parapsilosis* HC isolate could be correlated with the significantly decreased level of TAG. The very close relatedness between both the isolates suggests that one isolate was derived from another after the initial infection of the host.

## 1. Introduction

*C. parapsilosis* is an opportunistic yeast capable of causing systemic infections [1,2], mostly due to the colonization of prosthetic devices that are frequently associated with biofilm formation. This yeast is the second most isolated species (sp.) of the genus *Candida* [3,4], frequently associated with mortality in neonatal intensive care units [5,6,7,8,9]. Among the risk factors associated with *C. parapsilosis* infection, the application of a central venous catheter (CVC) is the most relevant [10,11].

The resistance to fluconazole (FLC), the most widely used antifungal drug among azole derivatives, can be caused by (i) up-regulation of the *ERG11* gene, (ii) up-regulation of the efflux transporter genes *CDR1* and *MDR1*, and (iii) point mutations in the *ERG11* and *ERG3* genes [12,13,14,15,16,17,18].

Several outbreaks of Y132F mutation-carrying *C. parapsilosis* (associated with the *ERG11* gene) have already been reported in many countries, such as Brazil, Turkey, Italy, and South Africa [19,20,21,22]. Combined with other single nucleotide polymorphisms (SNPs), such as R398I or K143R, Y132F substitution has been linked to increased FLC resistance [22,23,24]. This mutation was also previously found in azole-resistant *Candida albicans* [25,26]. Berkow et al. (2015) acknowledged that the variance in azole resistance between their tested isolates was high and the presence of these SNPs could not fully explained it [27]. Therefore, other mechanisms, such as those already described, or lipid composition can contribute to FLC resistance as well.

Information on the *C. parapsilosis* lipid profile is limited, but *C. albicans* shows a high similarity in many genes that have the same function [3]. Several studies have described imbalances in lipid homeostasis associated with FLC resistance in *C. albicans* [28,29]. Among relevant lipids, an accumulation of 14α-methyl-3,6-diol leads to intracellular toxicity and cell death [30,31] and fluctuations in phosphatidylglycerol (PG) are linked to azole resistance and cell wall integrity [32]. Free fatty acids (FFA) are an important growth factor also responsible for better biofilm formation in *C. parapsilosis*. Deletion of the *FAS2* gene (involved in FFA synthesis) suggests an impaired defence against macrophages with mutants more susceptible to oxidative stress [33].

In the previous study, two FLC-resistant *C. parapsilosis* isolates, namely one from CVC and the other from haemoculture (HC), originating from the same patient, were described in respect to their ability to form mixed biofilms with bacteria, and possible contribution of the *CDR1*, *MDR1* and *ERG11* gene regulation to FLC. Additionally, differences between both isolates based on whole genome sequencing (WGS) identified the R398I and Y132F substitutions associated with the *ERG11* gene were observed due to the following nucleotide substitutions, A395T and G1193T, respectively [12]. Moreover, two identical non-synonymous mutations (A622G leading to S208G and A910G leading to S304G) and one synonymous mutation (T1104C) were observed in the *ERG6* and *ERG9* genes, respectively, compared to standard strain *C. parapsilosis* CDC317 [12]. As both genes are involved in ergosterol (ERG) synthesis, it was supposed that mutations might also contribute to FLC resistance [34,35].

Secretion of hydrolytic enzymes has been associated with fungal virulence; primarily secreted aspartyl proteinases have been investigated and well documented [36,37]. In contrast, very little is known about the role of extracellular lipases. Only two enzymes have been identified in *C. parapsilosis* (coded by the *LIP1* and *LIP2* genes) [38]. Lip2 was described as an important virulence factor in *C. parapsilosis* as it promotes survival in macrophages and mitigates the inflammatory response [39].

The present study provides a detailed analysis of differences between two clinical isolates of *C. parapsilosis* originating from the same patient. It addresses the following: (i) differences between isolates in terms of virulence (possible relationship to the site of isolation; and (ii) a possible association between FLC resistance (Y132F and R398I mutations in the *ERG11* gene; the *ERG6* and *ERG9* genes) and lipid distribution.

## 2. Materials and Methods

### 2.1. Characterization of C. parapsilosis Isolates

Two previously described clinical isolates of *C. parapsilosis* [12]; one isolated from peripheral blood—*C. parapsilosis* HC, and the other from CVC—*C. parapsilosis* CVC, were used. Both isolates were resistant to FLC with minimal inhibitory concentration (MIC) higher than 256 µg/mL and were considered isogenic based on WGS analysis with various mutations relevant to resistance genes, including *ERG6* and *ERG9* [12].

*C. parapsilosis* CDC317, (kindly provided by Prof. J. Nosek, DrSc., Comenius University in Bratislava, Faculty of Natural Sciences, Dept. of Biochemistry, Bratislava, Slovakia) was used as the standard control strain in all experiments.

### 2.2. Formation of a 24 h Biofilm and XTT Reduction Assay

All strains were preserved in 1 mL of yeast extract peptone dextrose broth supplemented with 30% of sterile glycerol (Centralchem, Bratislava, Slovakia) at −80 °C (YPD broth, 1% yeast extract, Biolife, Milan, Italy; 2% mycological peptone, Lab M Limited, Buri, UK; 2% D-glucose, Centralchem, Bratislava, Slovakia). From this stock culture, 100 µL was added to 40 mL of YPD broth and cultivated at 37 °C overnight on a thermal shaker at 150 rpm (Multitrone Standard, Infors HT, Basel, Switzerland). The inoculum was plated on YPD agar (YPD broth with 2% of agar; Serva Electrophoresis GmbH, Heidelberg, Germany) plates and cultivated at 37 °C overnight to obtain single colonies. The next day, one colony was taken and resuspended in 40 mL of YPD broth and cultivated at 37 °C overnight with shaking at 150 rpm. The inoculum was later transferred to a 50 mL Falcon tube (Sarstedt AG & Co. KG, Nümbrecht, Germany) and centrifugated at 3000× *g*, 5 min, 15 °C (Universal 32R, Andreas Hettich GmbH & Co. KG, Tuttlingen, Germany). The supernatant was discarded, and the pellets were washed with 40 mL of 0.1 M phosphate saline buffer pH 7.2 (PBS, MP Biomedicals, LLC, Irvine, CA, USA) and centrifugated at 3000× *g*, 5 min, 15 °C. The washing step was repeated two more times under the same conditions. The supernatant was discarded, and the pellet was resuspended in 20 mL of PBS. The density of cells was calculated using a haemocytometer (Paul Marienfeld GmbH & Co. KG, Lauda-Königshofen, Germany) and set to 1 × 10^6^ cells/mL in RPMI-1640 medium without phenol red supplemented with 2% of D-glucose and buffered to pH 7.0 with 0.165 M MOPS. Biofilms were set up in a 96-well flat-bottom plate (TC Plate, Sarstedt AG & Co. KG, Nümbrecht, Germany) using 100 µL of 1 × 10^6^ cells/mL suspension. The plates were cultivated for 24 h at 37 °C in a thermal incubator (Thermostatic Cabinet, Lovibond, Tintometer GmbH, Dortmund, Germany). Biofilm was then evaluated for metabolic activity using XTT assay [40,41]. The experiment was performed in at least three parallel wells in two independent replicas. Values are represented as mean with standard deviations (SD).

### 2.3. Isolation of RNA

Two clinical isolates and the reference *C. parapsilosis* CDC317 were grown up to 16 h in 20 mL of YPD broth with/without sub-inhibitory concentration of FLC (2 µg/mL; Pfizer, New York, NY, USA) in an orbital shaker (Multitrone Standard, Infors HT, Bottmingen-Basel, Switzerland), at 180 rpm at 30 °C. Then, density was adjusted to OD_600_ 0.02 and *C. parapsilosis* strains were further cultivated in YPD broth with/without FLC (2 µg/mL) to reach the exponential phase (about 5 h). Then, the cultures were transferred to a new sterile 50 mL Falcon tube (Sarstedt AG & Co. KG, Nümbrecht, Germany) and centrifuged at 3000× *g*, 3 min at 15 °C (Universal 32R, Andreas Hettich GmbH & Co. KG, Tuttlingen, Germany). The supernatant was discarded, and cells were washed twice with PBS. After discarding the supernatants, 1 mL of PBS was added to resuspend the cells. Then, the yeasts were transferred to new microcentrifuge tubes and centrifuged at 8000× *g*, 2 min, 15 °C. Afterwards, the supernatants were discarded, and the isolation of RNA proceeded using the GeneJET RNA Purification Kit (Thermo Scientific, Waltham MA, USA) with the following modification: 200 µL of yeast lysis buffer was added to resuspend the pellet followed by a 60 min incubation at 30 °C. All further steps were conducted according to the manufacturer’s protocol. Eluted RNA was then purified with DNase I (Thermo Scientific, Waltham, MA, USA) and samples were stored at −80 °C or used immediately in a downstream application. To obtain cDNA for qPCR experiments, a cDNA synthesis kit was used (Maxima First Strand cDNA Synthesis Kit, Thermo Scientific, Waltham, MA, USA) according to the manufacturer’s instructions. Synthesized cDNA was stored at −20 °C or used immediately in qPCR.

### 2.4. Relative Gene Expression

Primers for the *ERG6*, *ERG9* and *LIP2* genes (designed by Dr. Adam Valček, Comenius University in Bratislava, Bratislava, Slovakia) and for the *ACT1* housekeeping gene [42] were synthesized by Metabion International AG, Planegg/Steinkirchen, Germany). The list of primers is summarized in Supplementary Data (Table S1). For initial confirmation via PCR, a thermal protocol was set up (initial denaturation at 95 °C for 1 min followed by 40 cycles of denaturation at 90 °C for 15 s, annealing at 58 °C for 1 min and extension at 72 °C for 1 min, followed by a final extension at 72 °C for 10 min). Gel electrophoresis was performed to confirm product lengths (2% agarose gel, 120 V, 90 min). Afterwards, a thermal protocol for 2-step qPCR (40 cycles of denaturation at 95 °C for 15 s and annealing at 58 °C for 1 min) was set up. For the reaction, HOT FIREPol^®^ EvaGreen^®^ qPCR Mix Plus (Solis BioDyne OÜ, Tartu, Estonia) and Mx3000P qPCR system (Agilent Technologies, Inc., Santa Clara CA, USA) was used. All data were analysed using the MxPro software provided by Agilent Technologies. Relative gene expression was calculated using the 2^ΔΔCq^ method [43]. *C. parapsilosis* CDC317 was set up as the control sample normalized to a value of 1.

Relative changes in expression of the *ERG6* and *ERG9* genes in CDC317 strain, and the isolates of HC and CVC after ~5 h incubation in the presence of a sub-inhibitory concentration of FLC (2 µg/mL) were compared to the non-treated samples of corresponding isolates set to 1. Any expression value ≥2 was considered as up-regulation. All experiments were performed in three parallel wells and six independent replicas. Values are represented as mean with SD.

### 2.5. Cultivation of Cells for Lipid and Fatty Acid Analysis

For lipid and fatty acid (FA) analysis, yeast cells were grown aerobically at 30 °C in YPD in Erlenmeyer flasks with cotton stoppers filled with medium up to 1/5 of flask volume. Next, cells were pre-cultured in YPD medium at 30 °C overnight and the resultant cultures were inoculated to the initial OD_600_ = 0.1 into fresh YPD medium with and without FLC (2 µg/mL). Cells were collected via centrifugation at 2500 × *g* for 5 min at the mid-logarithmic growth phase (OD_600_ between 0.8 and 1) and the pellet was washed once with distilled water and stored at −20 °C before lipid extraction.

### 2.6. Lipid Extraction and TLC Analysis of Neutral Lipids

Total lipids were extracted as described in [44]. Briefly, yeast cells corresponding to aliquot of 1 × 10^9^ cells were harvested via centrifugation and washed with distilled water. Briefly, 1 mL of chloroform:methanol (2:1, *v*/*v*, VWR International, Radnor, PA, USA) was added to the cell pellet and subsequently, the cells were disrupted by vortexing using glass beads (diameter 0.4 mm, BioSpec, Bartlesville, USA) 6 times for 1 min with cooling on ice between cycles. Lipids were extracted via incubation in chloroform/methanol/water (1:2:0.8, *v*/*v*) and subsequent adjustment of the proportion to (2:2:1.8, *v*/*v*) at room temperature (RT). The organic phase (lower phase) containing lipids was collected after centrifugation at 2500× *g* for 5 min and evaporated under a stream of nitrogen. Dry lipids were dissolved in 50 µL of chloroform and methanol (2:1, *v*/*v*). Briefly, 20 µL of lipid extracts corresponding to 3 × 10^8^ cells were separated via thin-layer chromatography (TLC) on silica gel TLC plates (Merck, Darmstadt, Germany) using a semiautomatic sample applicator (CAMAG Linomat 5, Muttenz, Switzerland). Neutral lipids were first separated in petroleum ether-diethyl ether-acetic acid (70:30:2, VWR International, Radnor, PA, USA). The TLC plate was developed to 2/3 of the plate in the first mobile phase, dried and further developed in the second mobile phase consisting of petroleum ether-diethyl ether (49:1; *v*/*v*) to the top of the plate [45].

To visualize spots corresponding to separated neutral lipids, TLC plates were dipped into a charring solution consisting of 5.16 g MnCl_2_.4H_2_O (Slavus s.r.o., Bratislava, Slovakia), 465 mL of water, 500 mL of methanol and 33 mL of concentrated sulfuric acid (Slavus s.r.o., Bratislava, Slovakia). Subsequently, TLC plates were incubated at 130 °C for 10 min. Individual peaks were identified by lipid standards run on the same plate. For semi-quantitative analysis, relative amounts of individual lipids were determined via densitometry at 600 nm (CAMAG TLC Scanner 3, Muttenz, Switzerland). The values represent the average of four independent experiments ± SEM. 

### 2.7. Extraction and Analysis of Sterols

Non-saponifiable lipids were extracted using the modified procedure of Breivik and Owades [46]. Aliquots of 1 × 10^9^ cells were suspended in 1 mL cold distilled water and cells were broken by vortexing with glass beads 6 times for 1 min with cooling on ice between cycles. After incubation in 3 mL of 60% KOH (*w*/*v*, Honeywell International Inc, Charlotte, NC, USA) in 50% methanol (*v*/*v*) for 2 h at 70 °C, lipids were extracted with 3 mL of *n*-hexane (VWR International, Radnor, PA, USA), organic (upper) phase was collected, and the remaining phase was re-extracted with 2 mL of *n*-hexane. Combined extracts were dried under a stream of nitrogen and lipid residue was dissolved in acetone (VWR International, Radnor, PA, USA). Sterols were analysed using reversed-phase high-performance liquid chromatography (HPLC) on the Agilent 1100 instrument equipped with Eclipse XDB-C8 column (Agilent Technologies, Inc., Santa Clara CA, USA), diode array detector (Agilent Technologies, Inc., Santa Clara CA, USA), and Corona Charged Aerosol Detector (Esa Biosciences, Inc., Chelmsford, MA, USA). Sterols were eluted at 40 °C with 95% methanol at the flow rate of 1 mL/min. Peaks were identified by retention times of individual lipid standards and verified by their characteristic UV spectra. Minor precursors represent the sum of 3 minor sterol intermediates, where each accounts for less than 2%. The values represent the average of 4 independent experiments ± SEM.

### 2.8. Fatty Acid Analysis

The FA composition of cellular lipids was determined using gas chromatography after FA trans-methylation according to [47]. Briefly, 1 mL of 10% methanolic HCl and 0.5 mL of dichloromethane (VWR International, Radnor, PA, USA) were added to 20 µL aliquot of total lipid extract corresponding to 1 × 10^8^ cells, followed by incubation at 60 °C for 6 h. The resultant fatty acid methyl esters (FAME) were extracted with 1.5 mL of saturated NaCl (Slavus s.r.o., Bratislava, Slovakia) solution and 1.25 mL of *n*-hexane. Subsequently, the tubes were centrifuged at 2500× *g* for 5 min, and the upper (hexane) layer was transferred to a glass vial. Analysis of FAME was performed by applying 1 μL aliquots to a gas chromatograph (GC) (GC2010Plus, Shimadzu, Japan) equipped with BPX70 capillary column (30 m × 0.25 mm × 0.25 µm, SGE Analytical Science, Australia) under temperature programming as described in [48]. Individual FAME were identified by comparison with the authentic standards of the C4−C24 FAME mixture (Supelco, Bellefonte, PA, USA). The values represent the average of three independent experiments ± SD.

### 2.9. Lipase Activity and Prediction of the Lip2 Protein Structure

Yeast cells were inoculated to initial OD_600_ = 0.1 into fresh YPD medium with and without FLC (2 µg/mL) and cultivated at 30 °C to reach the mid-logarithmic growth phase. Cells were collected via centrifugation at 2500× *g* for 5 min and the pellet was washed once with distilled water. Cells were suspended in 1 mL of 0.1M PBS pH 7.2 and disrupted with glass beads in FastPrep24 homogenizer (MP Biomedicals, Santa Ana, CA, USA) for 2 × 45 s, with 5 min cooling on ice between the runs. The homogenate was centrifuged at 20,000× *g* for 30 min at 4 °C to obtain the cytoplasmic extract. Total protein concentration in extracts was determined using the Bradford assay [49]. The lipase activity of yeast strains was determined via spectrophotometry using p-nitrophenyl butyrate (p-NPB, Biosynth, Berkshire, UK) as a substrate. The assay was performed in 200 µL of total volume, where 20 µL of the sample (80 µg of proteins) was added into 180 µL of substrate solution. The substrate solution consisted of 0.1 mL of solution A (20.9 mg of p-NPB dissolved in 10 mL absolute ethanol) mixed with 10 mL of solution B (0.1 M sodium-potassium phosphate buffer, pH 7.2 containing 0.1 M NaCl) [50]. Lipase activity was determined by measuring p-nitrophenol (pNP) absorbance at λ 405 nm (Multiskan GO, Thermo Scientific, Waltham, MA, USA) every 5 min in 60 min intervals at 37 °C released by protein extracts and calculated in pNP nmol. The values represent the average of three independent experiments ± SD.

The structure of the protein encoded by the *LIP2* gene in *C. parapsilosis* HC harbouring P80S substitution [12] was generated using the AlphaFold2 ColabFold online tool [51,52].

### 2.10. Statistical Analysis

The statistical analysis was performed using two-way ANOVA in GraphPad Prism 8 software (Graph Pad, San Diego, CA, USA). Sidak’s and Tukey’s multiple comparisons tests were used to determine significant differences between groups. Differences were considered to be significant at different *p*-values: *p* > 0.05 was considered non-significant, *p* < 0.05 (*) significant, *p* < 0.01 (**) very significant, *p* < 0.001 (***) highly significant, and *p* < 0.0001 (****) extremely significant.

## 3. Results

### 3.1. C. parapsilosis HC Demonstrated Higher Biofilm Metabolic Activity Compared to CVC after FLC Treatment

**Figure 1:**
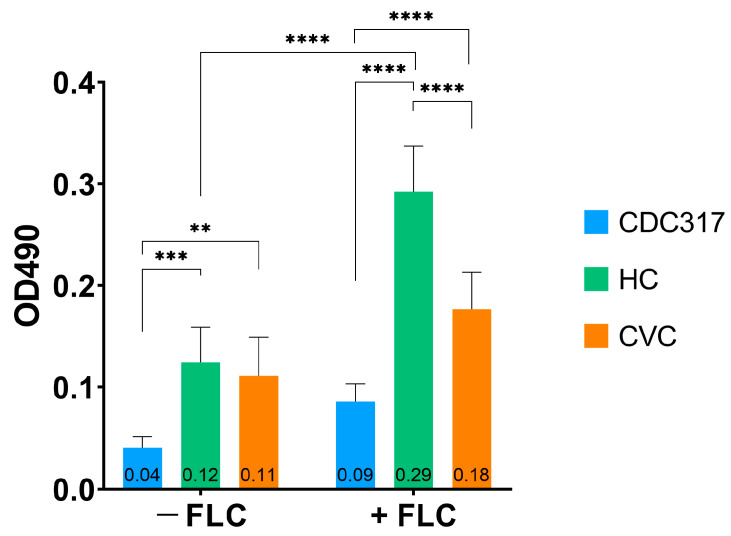
Metabolic activity of 24 h biofilms of *C. parapsilosis* CDC317, HC and CVC cultivated without (−FLC) and with a sub-inhibitory concentration of FLC (+FLC, 2 μg/mL). A *p* < 0.01 (**) was considered very significant; *p* < 0.001 (***) highly significant, and *p* < 0.0001 (****) extremely significant.

Figure 1 documents the metabolic activity of the biofilms of both clinical isolates (*C. parapsilosis* HC and CVC) and standard strain (CDC317). The biofilm metabolic activity was enhanced after exposure to a sub-inhibitory concentration of FLC (2 μg/mL) for all isolates; nevertheless, only for *C. parapsilosis* HC, the increase was significantly different (*p* < 0.001). Biofilms assembled by the clinical isolates (HC and CVC) either in the presence or absence of FLC exhibited significantly higher metabolic activity than CDC317.

### 3.2. Regulation of the ERG6 and ERG9 Genes Triggered by FLC

**Figure 2:**
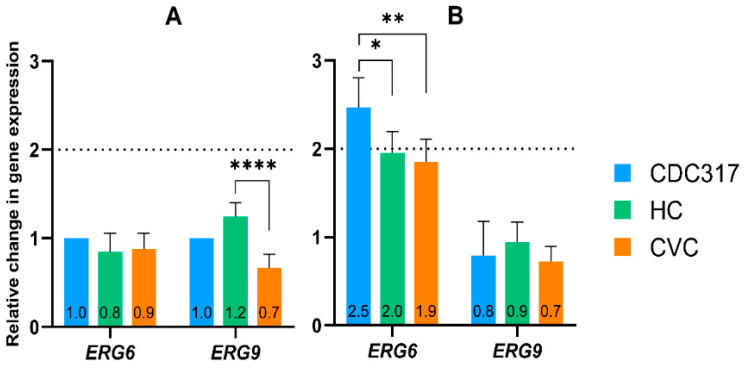
Relative change in the *ERG6* and *ERG9* gene expression before and after FLC in 3 *C. parapsilosis* strains. (**A**) The chart represents relative changes in isolates of *C. parapsilosis* HC and CVC compared to standard *C. parapsilosis* CDC317 that was set up as a control and normalized to the value of 1. (**B**) Relative changes expression determined after 5 h incubation in the presence of a sub-inhibitory concentration of FLC (2 µg/mL), non-treated samples of corresponding isolates were set to 1. A *p* < 0.05 (*) was considered statistically significant; *p* < 0.01 (**) very significant; *p* < 0.0001 (****) extremely significant.

Figure 2 summarizes a regulation of the *ERG6* and *ERG9* genes with and without FLC. Results showed that the expression of both genes in the natural planktonic state without FLC (Figure 2A) was not significantly affected compared to the control strain. After adding FLC, the *ERG6* gene was up-regulated in all tested isolates, and this up-regulation oscillated around 2-fold compared to the same strain without incubation with FLC (Figure 2B). Expression of the *ERG9* gene was not significantly changed in respect to FLC treatment suggesting that regulation of this gene was not involved in FLC resistance in tested *C. parapsilosis* isolates (Figure 2B).

### 3.3. C. parapsilosis HC and CVC Isolates Differ in Neutral Lipid Composition

**Figure 3:**
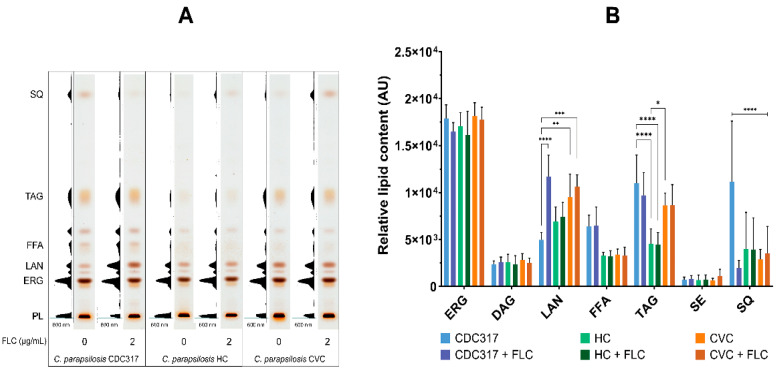
Neutral lipid profiles of *C. parapsilosis* standard strain CDC317 and HC and CVC clinical isolates. (**A**) TLC analysis of neutral lipids. The figure shows a representative result from 4 independent experiments. (**B**) Semi-quantitative analysis of lipids determined in AU (arbitrary units). List of determined lipids: PL—phospholipids; ERG—ergosterol; DAG—diacylglycerol; LAN—lanosterol; FFA—free fatty acids; TAG—triacylglycerols; SE—sterol esters; SQ—squalene. A *p* < 0.05 (*) was considered statistically significant; *p* < 0.01 (**) very significant; *p* < 0.001 (***) highly significant; *p* < 0.0001 (****) extremely significant.

Using TLC analysis, the neutral lipid pattern was estimated in all the strains in the absence and the presence of FLC (Figure 3). Control strain CDC317 cultivated with the sub-inhibitory concentration of FLC accumulated lanosterol (LAN), as expected, compared to untreated CDC317 cells. Interestingly, accumulation of LAN was observed in both resistant isolates cultivated in the absence of FLC and treatment with FLC did not significantly increase LAN accumulation (Figure 3A,B).

TLC analysis of lipids extracted from both clinical isolates (treated and untreated with FLC) also revealed a detectable, but non-significant drop in FFA compared to the control strain CDC317 (Figure 3A). With the drop in FFA, a significant decrease in levels of triacylglycerols (TAG) was observed in both tested strains. While CVC isolate showed slightly reduced TAG content (by 20%), a much substantial drop was determined in the HC isolate (by 60%) (Figure 3B).

In addition to neutral lipid composition analyses, PL were also analysed using TLC densitometry. However, no significant changes were observed in major PL (phosphatidylcholine—PC, phosphatidylethanolamine—PE, phosphatidylserine—PS, phosphatidylinositol—PI, and cardiolipin—CL).

*C. parapsilosis* HC and CVC were further analysed in terms of ERG biosynthetic intermediates (other than LAN) using HPLC. The observed accumulation of LAN in the absence of FLC in resistant clinical isolates (Table 1) confirmed the results from the TLC analysis. In both HC and CVC isolates, ergosta-5,7-dien-3β-ol was reduced compared to the control strain CDC317 regardless of treatment by FLC. In addition, decreased level of squalene (SQ) was detected in both untreated HC and CVC isolates. Apart from sterol intermediates, no significant differences in ERG content were observed in both isolates after FLC treatment.

### 3.4. FLC-Resistant C. parapsilosis Differ in FA Composition

**Figure 4:**
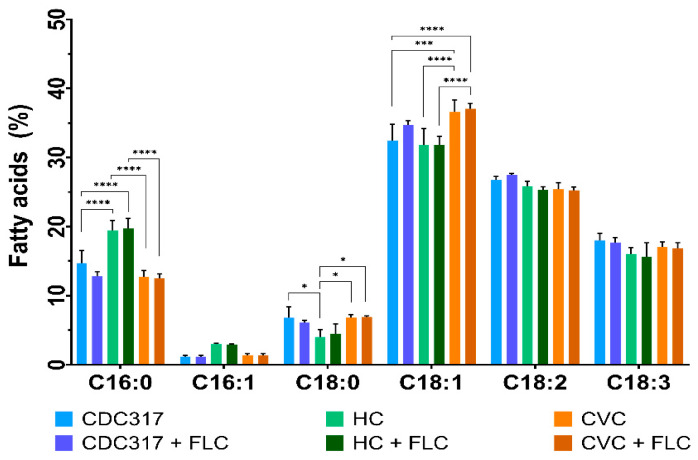
Fatty acid composition of *C. parapsilosis* strains of CDC317, HC and CVC. List of determined fatty acids: C16:0—palmitic acid; C16:1—palmitoleic acid; C18:0—stearic acid; C18:1—oleic acid; C18:2—linoleic acid; C18:3—α-linolenic acid. A *p* < 0.05 (*) was considered statistically significant; *p* < 0.001 (***) highly significant; *p* < 0.0001 (****) extremely significant.

Because of observed differences in the TAG of FLC-resistant isolates, FA composition was investigated via gas chromatography (Figure 4). In all isolates, the dominating FA were as follows: C18:1 (32–38% of total lipid content), C18:2 (25–27%), C18:3 (16–18%), C16:0 (12–20%), C18:0 (4–7%), and C16:1 (1–3%). FLC treatment mainly affected the control strain CDC317 and resulted in an elevated level of C18:1, a slight increase in C18:2 and a decrease in C16:0 and C18:0. There were observed differences in the FA composition between the two isogenic clinical isolates, HC and CVC. HC strain showed a significant increase in C16:0, a non-significant increase in C16:1 and a decrease in C18:0 compared to CVC and the control strain. On the other hand, the CVC isolate displayed a significantly elevated level of C18:1 compared to the HC isolate as well as the control strain.

To analyse changes in the FA composition, the degree of FA unsaturation was evaluated (Table 2). The saturated FA (SFA) comprised 19–24% and consisted of palmitic and stearic acid, with the HC isolate having the highest SFA content. In the case of monounsaturated FA (MUFA), the content was 33–38% and the amount of MUFA was elevated in the CVC isolate. No significant differences were observed in polyunsaturated FA (PUFA) content in both the HC and CVC isolates, but their levels were slightly decreased compared to the control strain.

### 3.5. HC Isolate Exhibits Elevated Lipase Activity

**Figure 5:**
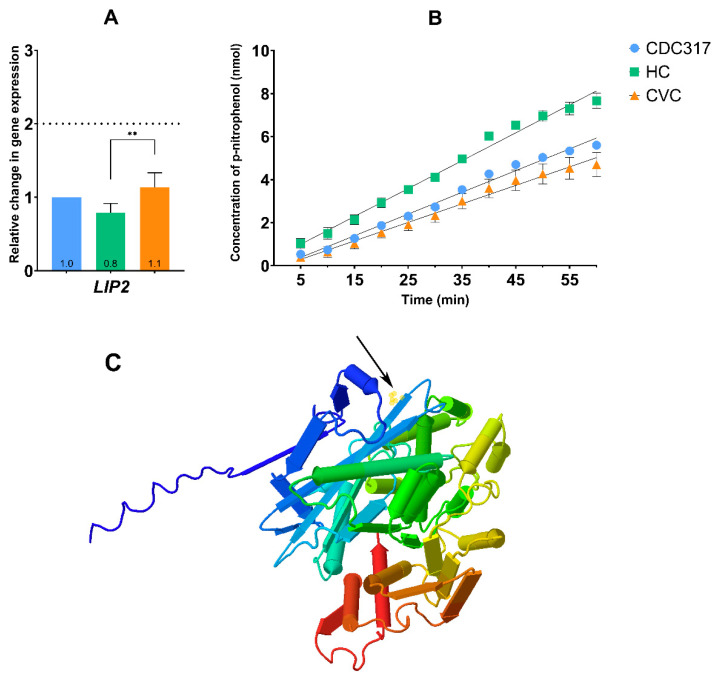
The *LIP2* gene expression and protein characterization of *C. parapsilosis* of CDC317, HC and CVC isolates. (**A**) Relative change in gene expression in *LIP2* gene A *p* < 0.01 (**) was considered very significant. (**B**) Enzymatic activity of lipase. (**C**) Predicted model of the Lip2 protein with an indicated area of PS80 (black arrow, yellow circles) substitution.

Decreased levels of TAG observed in both the clinical isolates of HC and CVC compared to the control strain CDC317 led to the investigation of the cellular lipase activity (Figure 4B). However, at first, the *LIP2* gene expression was estimated. Results did not show significant changes compared to the standard strain CDC317, but a slight difference was observed between the HC and CVC isolates (Figure 5A). As shown in Figure 5B, cytosolic extract from HC isolate exhibited elevated lipase activity compared to the CDC317 standard strain. A significant change was observed between CDC317 and HC from a 20 min time-point measurement. A similar difference was determined between the HC and CVC isolates from a 15 min time-point measurement.

Figure 5C shows the predicted structure of the Lip2 protein with the P80S substitution highlighted in yellow (black arrow). This substitution was observed only in the *C. parapsilosis* HC and exhibits elevated lipase activity (Figure 5B) possibly linked to the P80S substitution in Lip2.

## 4. Discussion

In the last decade, the incidence of *Candida parapsilosis* infections increased dramatically, therefore this representative of the genus *Candida* is of main interest to medical doctors and researchers [53,54,55]. This study expands previous work on two clinical isolates of *C. parapsilosis* recovered from a patient with a catheter-related bloodstream infection. Isolates of HC and CVC were previously subjected to WGS that confirmed some already mentioned differences in genome and both manifested high levels of resistance to FLC (>256 µg/mL). Previous results did not prove a contribution of the *CDR1* and *MDR1* efflux genes to FLC resistance, while the *ERG11* gene expression was significantly downregulated in the HC isolate compared to the CVC (0.9 and 0.5-fold, respectively, estimated to the control strain CDC317 set to 1). After treatment with a sub-inhibitory concentration of FLC, expression was only slightly increased in both *C. parapsilosis* (to 1.2 and 1.5-fold, for HC and CVC, respectively), compared to the same isolates without FLC. WGS revealed not only the presence of Y132F and R398I mutations in the *ERG11* gene but also the presence of non-synonymous mutations in the *ERG6* the *ERG9* genes [12]. Therefore, the regulation of both genes was further investigated in this study. Results showed that expression of *ERG6* in the presence of FLC increased, but still was under or about the threshold (2-fold) acceptable for a definition of regulation change compared to the same isolates in the absence of FLC. Therefore, regulation of tested genes does not seem important in terms of FLC resistance in both clinical isolates of *C. parapsilosis*. On the other hand, the downregulation of the *ERG11* gene and the presence of Y132F and R398I mutations was a motivation to analyse the impact of this gene on neutral lipid homeostasis in *C. parapsilosis* HC and CVC isolates.

Generally, the treatment of fungal cells with azoles affects the biosynthesis of ERG, a major fungal membrane sterol [35]. The ERG biosynthetic pathway is impaired by FLC through the inhibition of lanosterol 14-α-demethylase (Erg11), which is responsible for the conversion of LAN to 4,4-dimethylcholesta-8,14,24-trienol [56]. Berkow et al. (2015) [27] tested 35 FLC-resistant isolates of *C. parapsilosis*, while one of them harboured the Y132F SNP alone and the other ten isolates manifested the Y132F SNP along with R398I mutation similarly to tested HC and CVC isolates. Nine isolates from the study of Berkow et al. (2015) demonstrated increased levels of 14-αmethyl fecosterol, a sterol that was not detected in most of the other tested isolates and a higher level of LAN/obtusifoliol. They also suggested that the Y132F mutation alters the function of Erg11 in such a way that its function might be compromised, forcing a higher turnover through the enzymes encoded by the *ERG25/ERG26/ERG27* genes [27].

HPLC analysis provided in this study did not show significant differences in zymosterol or fecosterol content, but differences were present between LAN and SQ. Decreased levels of SQ detected in both isolates without FLC could suggest that squalene is faster consumed in both isolates compared to control strain to maintain LAN level. On the other hand, the level of SQ is usually low under standard conditions and, moreover, other factors, such as haem or oxygen supply can influence its accumulation in cells [57]. The increased quantity of unconverted LAN observed in both HC and CVC isolates reflects well with the level of the *ERG11* transcript [12]. It is possible that this increased level of unconverted LAN could be associated with the Y132F and R398I mutations in the *ERG11* gene, but a direct impact cannot be simply explained by these mutations themselves as CVC and HC isolates differed in the level of accumulated LAN. This observation indicates a contribution of some other factors to *ERG11* gene regulation.

In addition to the differential accumulation of LAN, changes in TAG level were also examined. A significant drop in TAG was observed in the HC isolate compared to both CVC and control strain CDC317. FA analyses revealed that the decrease in TAG for HC isolate affected the total FA composition. Indeed, HC isolate displayed increased levels of C16:0 and C16:1 and decreased level of C18:0 compared to both the CVC isolate and the control strain CDC317. The α-linolenic acid, in contrast to other studies [58,59], was highly enriched in the tested strains. This discrepancy in FA composition may be due to strain properties, but also due to differences in experimental conditions (growth phase of cells, and experimental conditions, such as composition of growth media, temperature, or aeration).

In *Saccharomyces cerevisiae*, TAG, SE, or PL represent the major pool of fatty acyl chains, accounting for most of the total FA-containing lipids in the cell. It is assumed that because of decreased level of TAG and low abundance of SE, changes in total FA composition in the HC isolate reflect changes in cellular PL. No changes detected in total FA composition in the CVC isolate may be due to the higher content of TAG compared to the HC isolate, which can buffer changes in the composition of FA in phospholipids. Taking into account PL analysis, no changes were observed in the level of major PL classes, but this does not exclude the possibility of change in FA composition in molecular species within individual PL as it was shown in a lipidomic study of *C. albicans* FLC tolerant clinical isolates [32].

Lipid metabolism is closely related to overall yeast metabolism. Measurement of metabolic activity might provide a brief overview of the dynamics of growth and virulence. Both the HC and CVC isolates were more active compared to the standard strain. When a sub-inhibitory concentration of FLC was added, the HC isolate showed higher metabolic activity compared to the CVC isolate. This might imply that the HC isolate was adapted to grow in the presence of FLC. Therefore, the growth of both isolates has been examined during 8 h and data were used to plot the growth curve of tested isolates cultivated with and without FLC (Supplementary Data, Figure S1). The CVC isolate did not show any major difference when cultivated with or without FLC. On the other hand, the HC isolate increased its biomass, even in the presence of FLC. The increased metabolic activity and growth in the presence of FLC observed for the HC isolate suggest that this isolate is better adapted to the host environment (selective advantage).

From the published WGS [12], the only distinction in genomes between the HC and CVC isolates was observed in the P80S mutation in the *LIP2* gene coding for lipase, an important virulence factor contributing to the dissemination of *Candida* in the host [60]. Results from qPCR did not show a significant change in the regulation of the *LIP2* gene. However, it is interesting that the HC isolate presented higher lipase activity than the CVC isolate. Since lipase activity is associated with TAG [37], decreased levels of TAG in the HC isolate could be associated with increased activity of this enzyme. Furthermore, P80S substitution might contribute to increased activity or production in HC isolate, as the predictive protein model suggested. A surface location of this substitution could lead to a selectively more efficient interaction with the environment. However, further studies are needed to confirm this hypothesis.

## 5. Conclusions

This study addressed two major questions; one was concerned with searching for differences between two isolates of *C. parapsilosis* originating from the same patient in terms of virulence and the second one was focused on a possible association between FLC resistance and lipid distribution. The already published WGS conducted on both HC and CVC isolates showed simultaneous mutations in the *ERG11*, *ERG6*, and *ERG9* genes. However, the regulation of the last two mentioned ones did not prove any impact on FLC resistance. Analysis of lipid metabolism showed some interesting results, such as higher accumulation of LAN in both resistant isolates regardless of FLC presence. Observed decreased levels in TAG affected the composition of total FA in the isolates. On the other hand, the direct impact of the *ERG11 mutations* on lipid/FA analysis has not been confirmed.

Summarizing the difference between both isolates, *C. parapsilosis* HC demonstrated a higher lipase activity that could be correlated with a significantly decreased level of TAG. The very close relatedness between both the isolates suggests that one isolate was derived from another after the initial infection of the host. *C. parapsilosis* CVC may have been colonized first, with continuous dissemination of dispersed yeast cells from the biofilm to the bloodstream, where it adapted better. This view supports results from the measurement of metabolic activity and growth curve in the presence of FLC. The probability that the CVC isolate is a revertant is less likely. A comparative analysis of both *C. parapsilosis* isolates revealed that despite their common ancestry, they can adapt differently according to their dependence on environmental conditions.

## Figures and Tables

**Table 1:** The spectrum of sterol composition (% of total sterols).

*C. parapsilosis*	Zymosterol	ERG	Ergosta-5,7-Dien-3β-ol	LAN	SQ	Minor Precursors
CDC317	0.8 ± 0.3	82.3 ± 1.4	1.9 ± 0.2	4.6 ± 0.9	6.8 ± 3.1	3.6 ± 0.3
CDC317 + FLC	0.7 ± 0.1	73.7 ± 1.6	1.8 ± 0.1	19.4 ± 1.2	0.8 ± 0.2	3.6 ± 0.2
HC	0.9 ± 0.1	82.9 ± 0.7	1.1 ± 0.1	8.6 ± 1.5	1.2 ± 0.7	5.3 ± 0.4
HC + FLC	0.9 ± 0.0	79.7 ± 0.6	1.2 ± 0.1	10.9 ± 0.9	2.1 ± 1.1	5.2 ± 0.6
CVC	0.6 ± 0.1	78.7 ± 1.2	1.1 ± 0.1	12.9 ± 1.8	2.0 ± 0.7	4.7 ± 0.4
CVC + FLC	0.6 ± 0.3	74.9 ± 1.4	1.1 ± 0.1	17.2 ± 1.8	1.5 ± 0.8	4.6 ± 0.5

ERG—ergosterol; LAN—lanosterol; SQ—squalene; FLC—fluconazole.

**Table 2:** The UFA/SFA ratio and % of SFA, MUFA and PUFA.

*C. parapsilosis*	C18:1/C18:0	UFA/SFA	SFA (%)	MUFA (%)	PUFA (%)
CDC317	5.0 ± 1.3	3.7 ± 0.7	21.5 ± 2.9	33.6 ± 1.8	44.8 ± 1.3
CDC317 + FLC	5.7 ± 0.4	4.3 ± 0.3	18.9 ± 0.9	35.9 ± 0.5	45.2 ± 0.9
HC	8.5 ± 2.5	3.3 ± 0.4	23.4 ± 2.1	34.8 ± 2.0	41.9 ± 0.1
HC + FLC	7.6 ± 2.5	3.2 ± 0.5	24.2 ± 2.4	34.8 ± 1.2	41.0 ± 2.1
CVC	5.3 ± 0.5	4.1 ± 0.3	19.6 ± 1.1	38.0 ± 1.2	42.5 ± 0.1
CVC + FLC	5.4 ± 0.3	4.2 ± 0.2	19.4 ± 0.7	38.4 ± 0.5	42.1 ± 0.4

C18:0—stearic acid; C18:1—oleic acid; MUFA—monounsaturated fatty acids; PUFA—polyunsaturated fatty acids; SFA—saturated fatty acids; UFA—unsaturated fatty acids.

## Data Availability

https://zenodo.org/record/7728161#.ZA7_ux-ZPIU, accessed on 13 March 2023. doi: 10.5281/zenodo.7728161.

## Supplementary Materials

The following supporting information can be downloaded at: https://www.mdpi.com/article/10.3390/cells12121579/s1, Table S1. Oligonucleotide sequences used in the study; Supplementary Figure S1. Growth curves of *C. parapsilosis*, CDC317, HC and CVC with (samples marked as +F) and without (samples marked as—F) presence of 2 µg/mL of FLC.
