# Supplementary material for: Comparative Analysis of Two Candida parapsilosis Isolates Originating from the Same Patient Harbouring the Y132F and R398I Mutations in the ERG11 Gene
Source: Cells. 2023 Jun 7;12(12):1579. doi: 10.3390/cells12121579 (PMC10296954; doi:10.3390/cells12121579)
Supplement: Supplementary file 1 [file cells-12-01579-s001.zip › cells-2311022-supplementary.pdf]

Article

Comparative analysis of two *Candida parapsilosis* isolates from the patient with a central venous catheter harbouring the Y132F and R398I mutations in the *ERG11* gene

Matúš Štefánek [1], Martina Garaiová [2], Adam Valček [3], Luisa Jordao [4] and Helena Bujdáková [1,*]

1 Department of Microbiology and Virology, Faculty of Natural Sciences, Comenius University in Bratislava, 842 15 Bratislava, Slovakia; stefanek8@uniba.sk, valcekam@gmail.com, helena.bujdakova@uniba.sk,
2 Institute of Animal Biochemistry and Genetics, Centre of Biosciences, Slovak Academy of Sciences, Dúbravska cesta 9, 840 05 Bratislava, Slovakia; martina.garaiova@savba.sk
3 Research and Development Unit, Department of Environmental Health, National Institute of Health Dr Ricardo Jorge, 1649-016 Lisboa, Portugal; maria.jordao@insa.min-saude.pt
* Correspondence: helena.bujdakova@uniba.sk; Tel.: +421 2 9014 9436

Table S1. Oligonucleotides sequences used in the study.

Name		Sequence	Reference
ACT1	forward	CGAACGTGGTTACGGTTTCT	Neji *et al.*, 2017 [42]
	reverse	TGACCATCTGGCAATTCGTA	
ERG9	forward	ACTTCGAGATCATTTGCGGC	This study
	reverse	TCCTCTCAACAATGGCACCT	
ERG6	forward	GTACAGTCGTGAAGTTGCCG	This study
	reverse	ATCCAACAGCTTCCGTGGTA	
LIP2	forward	CACGGTACCTTGGATGCAAT	This study
	reverse	ATCCAAGTCAAAGCAGCTGG	

Construction of growth curve

The growth of all strains was characterized by growth curve measured for every h on a microplate reader (MRX II Microplate reader, Dynex Technologies, Chantilly, VA, USA) at OD600 for total 8 h. From overnight culture grown at YPD agar plate at 30 °C, a starting inocula (OD_{600} = 0.02) were set up in 20 mL of YPD broth and cultivated on a thermal shaker (150 rpm; Thermostatic cabinet, Lovibonds, Biosan, Riga, Latvia) at 30 °C. The growth of HC isolate was boosted in the presence of FLC apart from CDC317 and CVC, starting from 4-h mark with higher differences observed in subsequent measurements. The highest grow rate was observed in *C. parapsilosis* HC in the presence of FLC.

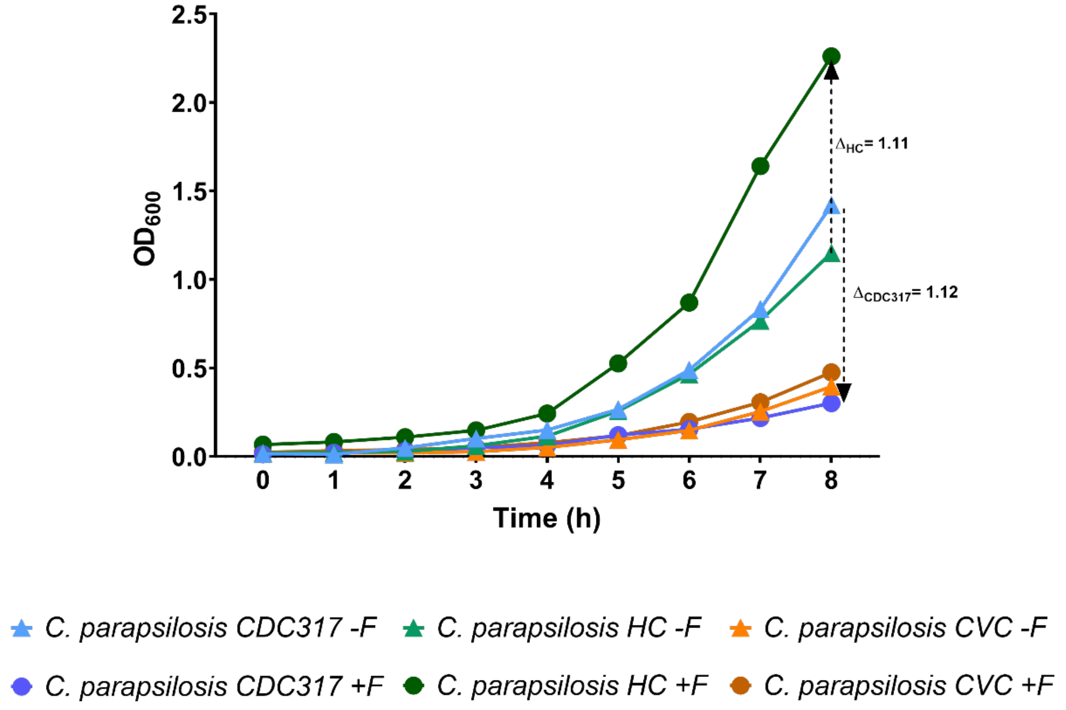

Supplementary Figure S1. Growth curves of *C. parapsilosis*, CDC317, HC and CVC with (samples marked as +F) and without (samples marked as -F) presence of 2 μg/mL of FLC.
